# Nitric oxide synthase-mediated early nitric oxide burst alleviates water stress-induced oxidative damage in ammonium-supplied rice roots

**DOI:** 10.1186/s12870-019-1721-2

**Published:** 2019-03-20

**Authors:** Xiaochuang Cao, Chunquan Zhu, Chu Zhong, Junhua Zhang, Lianghuan Wu, Qianyu Jin, Qingxu Ma

**Affiliations:** 1 State Key Laboratory of Rice Biology, China National Rice Research Institute, No. 359 Tiyuchang Road, Hangzhou Zhejiang, 310006 People’s Republic of China; 2 Ministry of Education Key Laboratory of Environmental Remediation and Ecosystem Health, College of Environmental and Resource Sciences, Zhejiang University, Hangzhou, 310058 China

**Keywords:** Ammonium, Nitric oxide, Nitric oxide synthase, Oxidative damage, Antioxidant enzymes, Water stress

## Abstract

**Background:**

Nutrition with ammonium (NH_4_^+^) can enhance the drought tolerance of rice seedlings in comparison to nutrition with nitrate (NO_3_^−^). However, there are still no detailed studies investigating the response of nitric oxide (NO) to the different nitrogen nutrition and water regimes. To study the intrinsic mechanism underpinning this relationship, the time-dependent production of NO and its protective role in the antioxidant defense system of NH_4_^+^- or NO_3_^−^-supplied rice seedlings were studied under water stress.

**Results:**

An early NO burst was induced by 3 h of water stress in the roots of seedlings subjected to NH_4_^+^ treatment, but this phenomenon was not observed under NO_3_^−^ treatment. Root oxidative damage induced by water stress was significantly higher for treatment with NO_3_^−^ than with NH_4_^+^ due to reactive oxygen species (ROS) accumulation in the former. Inducing NO production by applying the NO donor 3 h after NO_3_^−^ treatment alleviated the oxidative damage, while inhibiting the early NO burst by applying the NO scavenger 2-(4-carboxyphenyl)-4,4,5,5-tetramethylimidazoline-1-oxyl-3-oxide (c-PTIO) increased root oxidative damage in NH_4_^+^ treatment. Application of the nitric oxide synthase (NOS) inhibitor N(G)-nitro-*L*-arginine methyl ester(L-NAME) completely suppressed NO synthesis in roots 3 h after NH_4_^+^ treatment and aggravated water stress-induced oxidative damage. Therefore, the aggravation of oxidative damage by L-NAME might have resulted from changes in the NOS-mediated early NO burst. Water stress also increased the activity of root antioxidant enzymes (catalase, superoxide dismutase, and ascorbate peroxidase). These were further induced by the NO donor but repressed by the NO scavenger and NOS inhibitor in NH_4_^+^-treated roots.

**Conclusion:**

These findings demonstrate that the NOS-mediated early NO burst plays an important role in alleviating oxidative damage induced by water stress by enhancing the antioxidant defenses in roots supplemented with NH_4_^+^.

**Electronic supplementary material:**

The online version of this article (10.1186/s12870-019-1721-2) contains supplementary material, which is available to authorized users.

## Background

As human population and global climate change increase, drought stress is becoming a major abiotic factor limiting crop growth and yield. Plants have evolved several strategies to contend with water stress. These include morphological, physiological, and molecular adaptations [1–3]. As an important signaling molecule in various physiological functions like seed germination, floral transition, stomatal movement, leaf senescence, and yield development, nitric oxide (NO) has gained increasing attention since the 1980s [4–6]. Certain plant responses and adaptations to abiotic stresses also involve NO, and sufficient data indicate that NO mediates plant responses to various stimuli including drought [7], salt [8], and metal toxicity [9] stresses, thereby enhancing plant stress tolerance and survival.

Water deficits simultaneously increase endogenous NO and reactive oxygen species (ROS) production in plants [7, 10]. The accumulation of ROS in water-stressed plants impairs the function of biochemical processes, damages organelles, and ultimately results in cell death [11]. A combination of pharmacological analysis and transgenic technology has indicated that NO induces antioxidant activity and alleviates water stress in plants in several ways: (1) It limits ROS accumulation and ROS-induced cytotoxic activity by inhibiting the ROS-producer nicotinamide adenine dinucleotide phosphate oxidase via *S*-nitrosylation [12]. (2) It reacts with ROS (e.g. O_2_^.-^) to generate transient ONOO^−^, which is then immediately scavenged by other cellular processes [13]. (3) It induces the expression of genes coding for antioxidant enzymes, such as superoxide dismutase (SOD), ascorbate peroxidase (APX), and glutathione reductase (GR), and may increase enzyme activity, thereby reducing lipid peroxidation under water stress [14]. (4) It helps maintaining high vacuolar concentrations of osmotically active solutes and amino acids like proline [15]. (5) It acts as a downstream abscisic acid (ABA) signal molecule and participates in “ABA-H_2_O_2_-NO-MAPK” signal transduction processes, and thus increases plant antioxidant ability [16]. Therefore, endogenous NO production may enhance plant antioxidant capacity and help plant cells survive under various types of stress.

However, NO has biphasic properties on plants. The duality of its effects depends on stress duration and severity, and on the cell, tissue, and plant species [17]. At low concentration or in the early stage of abiotic stress, NO participates in important functions in higher plants through its involvement in physiological and stress-related processes (as described above). Some authors demonstrated that NO synthesis slightly increased in roots subjected to < 10 h water deficit, but was significantly up-regulated after prolonged drought (≥17 h) [18, 19]. Under severe or protracted stress, NO overproduction in plants can shift the cellular stress status from oxidative stress to severe nitrification stress, finally damaging proteins, nucleic acids, and membranes [13, 20]. Protein tyrosine nitration is considered a good marker to evaluate the process of nitrosative stress under various abiotic stresses [21]. Excess NO can also act synergistically with ROS, resulting in nitro-oxidative stress and eliciting undesirable toxic effects in plant cells [7]. Liao et al. [22] argued that the ability of endogenous or exogenous NO production in plants to alleviate oxidant damage was dose-dependent. Therefore, determining the instantaneous plant NO content under drought stress may not completely reflect the specific role of NO in drought tolerance.

In higher plants, nitrate reductase (NR) and nitric oxide synthase (NOS) are the two key enzymes for NO production [4, 23]. Moreover, NR-dependent NO production occurs in response to pathogen infection [24], aluminum [25], freezing [26], and drought [27]. For a long time, although NOS-like activity had been detected in plants, the gene(s) encoding NOS protein in higher plants remained to be identified [28]. Recently, some authors demonstrated that mammalian NOS inhibitors suppress NO production in response to various stimuli in plants [22, 29], suggesting that an arginine-dependent NOS activity may also occur in plants. Overexpression of rat neuronal NO synthase in plants increased their tolerance to drought stress, also demonstrating the importance of NOS-mediated NO production in tolerance of water deficits [30]. Arasimowicz-Jelonek et al. [18, 19] applied the NO donor sodium nitroprusside (SNP) and *S*-nitrosoglutathione (GSNO) to water-stressed cucumbers and demonstrated that both NR and NOS participated in drought tolerance. Despite increasing knowledge on NO-mediated plant functions, NO origins and signaling in response to prolonged stress and their regulation in plant drought tolerance remain poorly understood.

Ammonium (NH_4_^+^) and nitrate (NO_3_^−^) are the two primary N sources for plants. It is known that the negative effects of drought stress on plant development can be more effectively alleviated by NH_4_^+^ than NO_3_^−^ nutrition, as evaluated by plant growth, physiological characteristics, and gene expression levels [2, 31, 32]. NO has a key role in the acclimation of plants to water stress. Nevertheless, information on the dynamic changes in NO production and its role in drought acclimation in plants supplied with NO_3_^−^ or NH_4_^+^ during the early stage of water stress is scarce. In the present study, variations in endogenous NO production were monitored in roots supplied with these two N nutrition supplements during water stress. The specific role and origin of the endogenous NO produced were investigated using pharmacological methods. The present study revealed that an early NO burst is crucial for alleviating the water stress-induced oxidative damage through enhancement of antioxidant defenses in roots of NH_4_^+^-supplied plants. Further analyses demonstrated that this early NO burst might be triggered by NOS-like enzyme.

## Results

### Plant growth and physiological characteristics

**Fig. 1:**
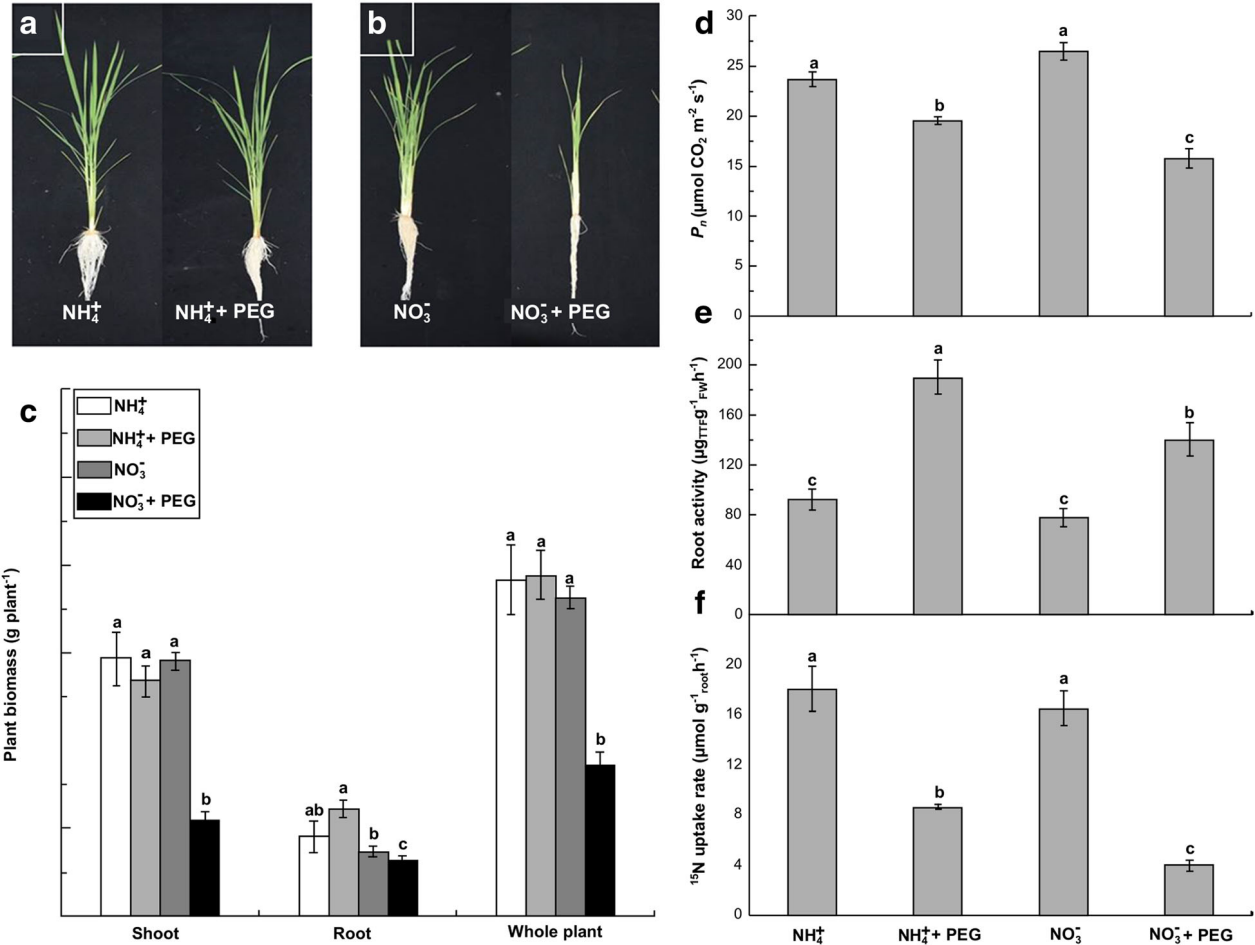
(**a**), (**b**) and (**c**) Response of rice agronomic characteristics and biomass in NH_4_^+^- and NO_3_^−^-supplied rice to water stress stimulated by 10% PEG after 21 days. (**d**) Effects of water stress on leaf photosynthesis in NH_4_^+^- and NO_3_^−^-supplied rice after 21 days. (**e**) Effects of water stress on root activity in NH_4_^+^- and NO_3_^−^-supplied rice after 21 days. (**f**) Effects of water stress on root ^15^N-labeled uptake rate in NH_4_^+^- and NO_3_^−^-supplied rice after 21 days. Rice leaf photosynthesis, root activity and ^15^N uptake rate was determined as described in Additional file 4: Method S1. Values represent means ± standard error (SE) (*n* = 6). Different letters refer to significant differences at *P* < 0.05. TTF: triphenylformazane; FW: fresh weight

Growth- and physiology-related parameters, such as biomass, net photosynthetic rate (*P*_n_), and root N uptake rate in rice seedlings supplied with different N sources were negatively and differently influenced by water stress (Fig. 1a–f). After 21 days of water stress (as a result of polyethylene glycol [PEG] treatment), root, shoot and total biomass were significantly decreased by 14.1, 62.1 and 52.4% in treatment with NO_3_^−^ and PEG, compared to its non-water stress treatment (NO_3_^−^ treatment, as the control treatment) (Fig. 1b, c). However, these values were not significantly affected in NH_4_^+^ with PEG treatment. Water stress also reduced leaf (*P*_n_) and root ^15^N uptake rate in the NO_3_^−^-treated plants by 40.4 and 76.1% (*P* < 0.05) in relation to non-water-stress-treated plants, but that of NH_4_^+^-treated plants was reduced by 17.3 and 52.2% (Fig. 1d, f). In contrast, root activity under water stress was increased by 106.2 and 79.6% in the NO_3_^−^-treated and NH_4_^+^-treated plants, respectively. Thus, it seems that NH_4_^+^-supplied rice seedlings can alleviate PEG-induced water stress more effectively than NO_3_^−^-supplied rice seedlings.

### Root endogenous NO production and histochemical analyses of oxidative damage

**Fig. 2:**
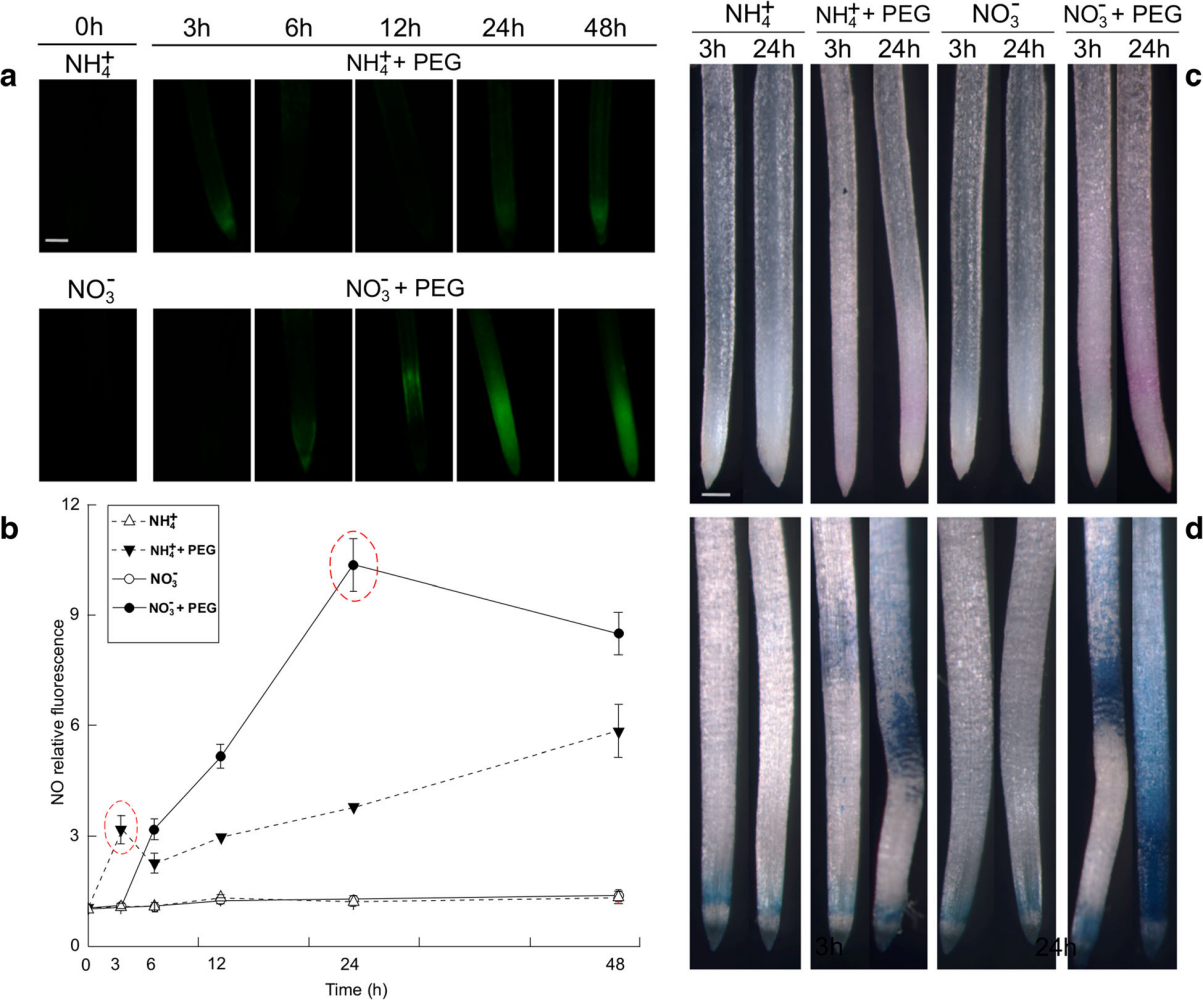
Time-dependent endogenous nitric oxide (NO) production and histochemical detection of oxidative damage in the root apices of NH_4_^+^- and NO_3_^−^-supplied rice seedlings under water stress. (**a**) Detection of NO fluorescence using DAF-FM DA staining and a fluorescence microscope. NO generation is indicated by green fluorescence. Bar = 300 μm. (**b**) NO production is expressed as relative fluorescence. To detect the NO production time course, seedling roots exposed to 10% PEG were collected at 0, 3, 6, 12, 24, and 48 h. (**c**) and (**d**) Histochemical detection of the aldehydes derived from lipid peroxidation and Evans blue uptake in root apices of rice seedlings under water stress. Rice seedlings were either untreated or subjected to 3 or 24 h of water stress, respectively. Roots were stained with Schiff’s reagent (**c**) and Evans blue (**d**), and then immediately photographed under a Leica S6E stereomicroscope (Leica, Solms, Germany). Red/purple indicates the presence of lipid peroxidation detected with Schiff’s reagent. Bar = 1 mm. Endogenous NO concentrations and histochemical detection of oxidative damage in the root are given. In Fig. 2b, the red dotted oval represents the high endogenous NO production in the NH_4_^+^- and NO_3_^−^-supplied rice, respectively. Values represent means ± standard error (SE) (*n* = 6). Different letters refer to significant differences at *P* < 0.05. Con indicates control treatment for each N nutrition, i.e., plants receiving non-water stress

To investigate whether NO participates in water stress acclimation, endogenous NO levels in roots were monitored with the NO-specific fluorescent probe diaminofluorescein-FM diacetate (DAF-FM DA) [25]. Significant differences in endogenous NO production were observed in roots after 48 h of water stress (Fig. 2a). In the NH_4_^+^ or NO_3_^−^-treated plants, NO production was relatively stable and varied little between the two N nutritions (Fig. 2b). In contrast, water stress significantly induced endogenous NO production 3 h after the roots received NH_4_^+^. However, endogenous NO gradually increased only after 6 h in the NO_3_^−^ treatment. Relative fluorescence indicated a significant early burst of NO at 3 h of water stress in the NH_4_^+^ with PEG treatment relative to the NH_4_^+^ treatment (Con). The NO level in the seedlings treated with NH_4_^+^ was 2.92 times higher than that of NO_3_^−^-treated plants. Nevertheless, NO in the NO_3_^−^-treated seedlings was 2.72 times higher than in NH_4_^+^-treated plants after 24 h of water stress (Fig. 2b).

**Fig. 3:**
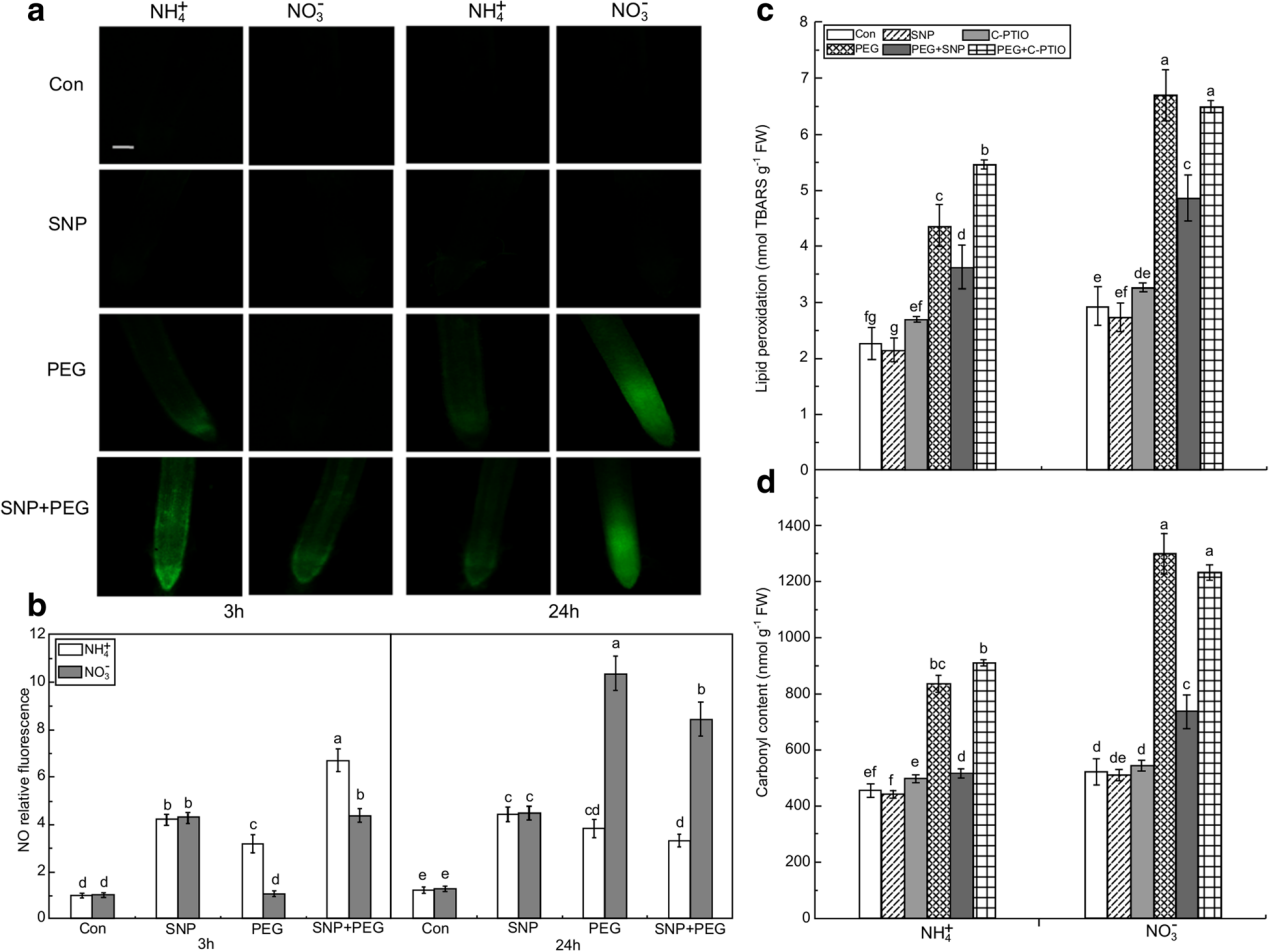
Responses of endogenous nitric oxide (NO) concentrations and oxidative damage to NO donor (SNP) or NO scavenger (c-PTIO) in root apices under the non-water stress (Con) or water stress conditions. (**a**) Photographs of NO production after SNP application. Bar = 300 μm. (**b**) NO production expressed as relative fluorescence. Rice seedlings were either untreated or treated with SNP under water stress. After 3 h and 24 h of treatment, root tips were loaded with 10 μM DAF-FM DA and NO fluorescence was imaged after 20 min using a fluorescence microscope. Endogenous NO concentrations in root are displayed. The lipid peroxidation (**c**) and carbonyl concentration (**d**) in rice roots represent the oxidative damage. In the c-PTIO and PEG + c-PTIO treatments, the rice seedlings were pretreated with NO scavenger (c-PTIO) for 3 h followed by non-water stress or water stress. After 3 h, the contents of MDA representing lipid peroxidation and carbonyl group in rice seedling roots were determined. Values represent means ± standard error (SE) (*n* = 6). Different letters indicate significant differences at *P* < 0.05. Con indicates control treatment for each N nutrition, i.e., plants receiving non-water stress. FW: fresh weight; TBARS: thiobarbituric acid reactive substances

Histochemical visualization by Schiff’s reagent and Evans blue staining showed that water stress caused severe oxidative damage to the plasma membrane and cell death in the roots of the plants receiving NO_3_^−^, whereas the damage was far less pronounced in the seedlings given NH_4_^+^ (Fig. 2c, d). The following analysis of the malondialdehyde (MDA) and carbonyl concentrations also confirmed that water stress induced more severe lipid peroxidation in the roots of NO_3_^−^-treated than in the roots of NH_4_^+^-treated seedlings (Fig. 3c, d).

### Effects of the NO donor on root NO production and oxidative damage

To determine the role of NO in water stress tolerance, the NO donor SNP was used to simulate NO production. Pre-experimentation with various SNP concentrations (0–100 μM) was performed to quantify the efficacy of SNP against root oxidative damage. As shown in Additional file 1: Figure S1, root oxidative damage induced by water stress was significantly alleviated by ≤20 μM SNP. However, the remedial effect of SNP on root oxidative damage was reversed at higher application doses (≥ 40 μM), suggesting that high SNP or NO contents are toxic to root growth. Therefore, 20 μM SNP was used in the NO donor experiment conducted in the present study. After 3 h of water stress, SNP application significantly increased root NO fluorescence intensity for both N nutrition. At 3 h, the NO production levels were ~ 2.05 and 3.85 times higher in the SNP + PEG-treated roots of the seedlings receiving NH_4_^+^ and NO_3_^−^, respectively, than in the PEG-treated roots of Con plants (Fig. 3a, b). However, this phenomenon was not observed after 24 h of water stress.

**Fig. 4:**
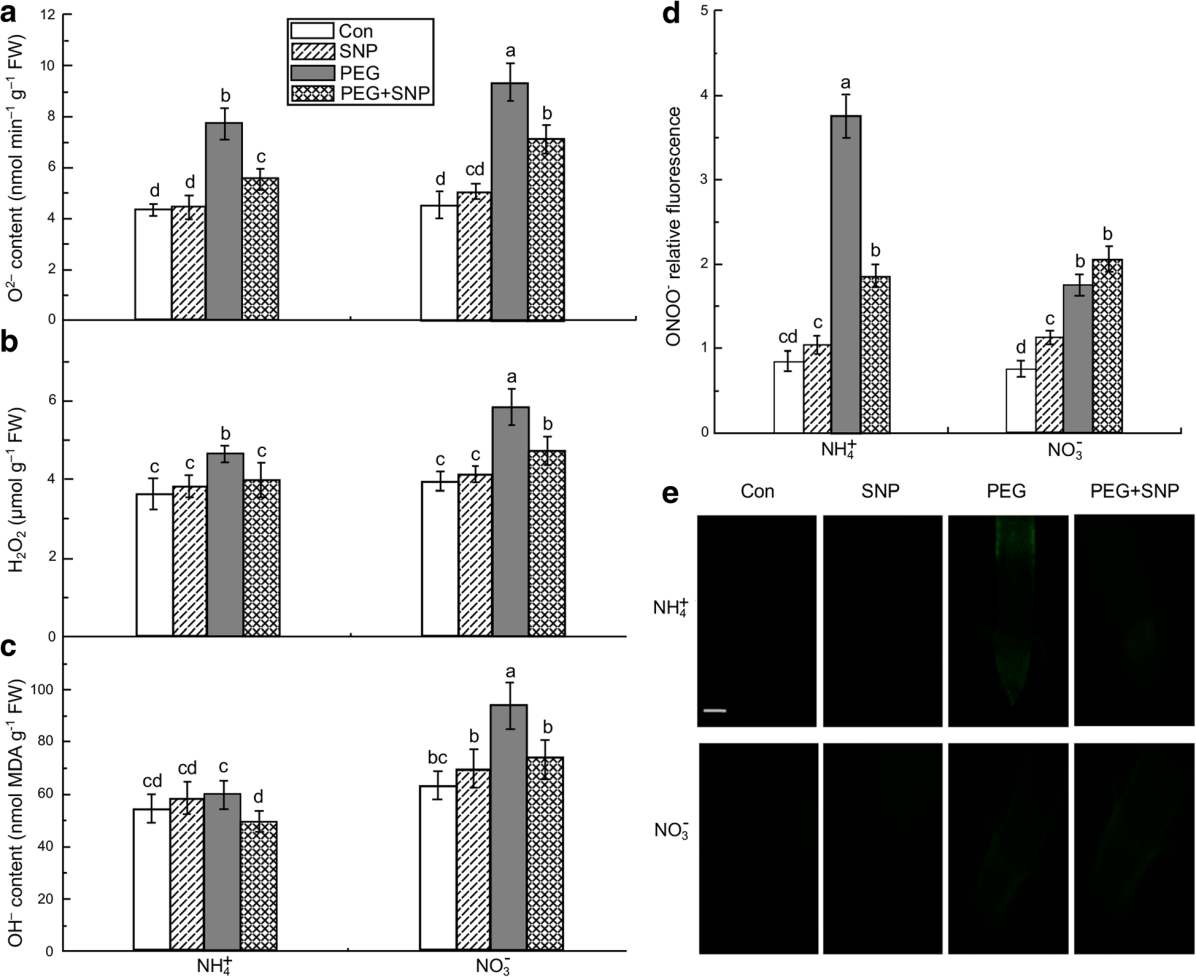
ROS and ONOO^−^ accumulation in root apices of rice seedlings treated with NO donor (SNP) and either receiving non-water stress (Con) or subjected to water stress using PEG. After 3 h, O_2_^.-^ (**a**), H_2_O_2_ (**b**), and OH^−^ (**c**) levels in rice seedlings roots were measured by spectrophotometry. ONOO^-^ production expressed as relative fluorescence (**d**). The accumulation of ONOO^-^ was detected with 10 μΜ aminophenyl fluorescein (**e**), Bar =3 00 μm. Fluorescence images and relative fluorescence intensity were analyzed as described in Fig. 2 for NO determination. Values represent means ± standard error (SE) (*n* = 6). Different letters indicate significant differences at *P* < 0.05. Con indicates control treatment for each N nutrition, i.e., plants receiving non-water stress. FW: fresh weight

After 3 h of water stress, ROS (O_2_^.-^, H_2_O_2_, and OH^−^) levels were increased in the roots of both the NH_4_^+^ + PEG- and NO_3_^−^ + PEG-treated seedlings in relation to their Con seedlings. Under water stress, the O_2_^.-^, H_2_O_2_, and OH^−^ in the roots given NH_4_^+^ and NO_3_^−^ increased by 78.1 and 107.3%, 28.3 and 47.8%, and 10.6 and 48.4%, respectively (Fig. 4a–c). Root MDA and carbonyl contents were ~ 1.28 and 1.4 times higher in the plants receiving NO_3_^−^ + PEG than in Con plants, respectively. In turn, MDA and carbonyl levels were significantly higher in NO_3_^−^ + PEG-treated plants than in plants given NH_4_^+^ + PEG (Fig. 3c, d). Water stress induced higher root ONOO^−^ in the NH_4_^+^-treated plants than in the NO_3_^−^-treated seedlings (Fig. 4d), and exogenous NO application significantly reduced water stress-induced >ROS (O_2_^.-^ and H_2_O_2_) accumulation and oxidative damage (as reflected by MDA and carbonyl) in both N nutritions (Figs. 3, 4).

To determine whether the alleviation of water stress-induced oxidative damage by SNP was related to NO production, the NO scavenger c-PTIO was applied to the plants. After pretreatment with 100 μM c-PTIO for 3 h, the alleviation of root oxidative damage induced by SNP application under water stress was reversed (Fig. 3c, d). Depletion of endogenous NO by c-PTIO significantly aggravated root oxidative damage in NH_4_^+^-treated plants but had no significant effect on the NO_3_^−^-treated plants, in relation to that observed in PEG-treated plants. Therefore, the water stress-induced early NO burst observed in the NH_4_^+^-treated plants alleviates root oxidative damage by reducing ROS, such as O_2_^.-^ and H_2_O_2_.

### Source of endogenous NO

**Fig. 5:**
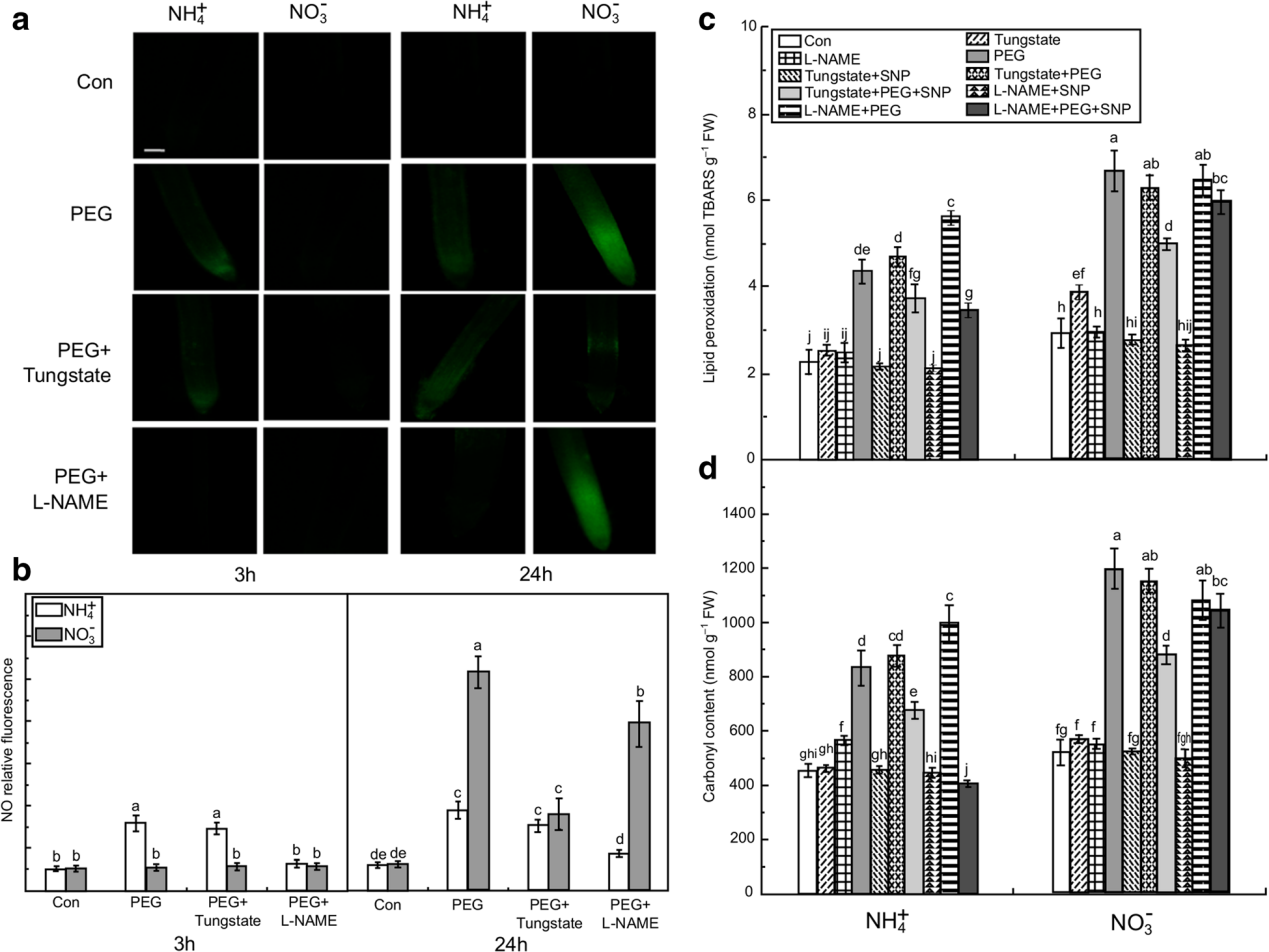
Effects of NR inhibitor (tungstate) and NOS inhibitor (L-NAME) on NO content and oxidative damage in root apices of rice seedlings. Rice seedlings were pretreated with NR inhibitor (100 μM tungstate) or NOS inhibitor (100 μM L-NAME) for 3 h, and then subjected to water treatment. (**a**) NO fluorescence. Bar = 300 μm. (**b**) NO production expressed as relative fluorescence. The contents of MDA representing lipid peroxidation (**c**) and carbonyl group (**d**) in rice seedling roots were measured after 3 h of water treatment following tungstate or L-NAME pretreatment. Values represent means± standard error (SE) (*n* = 6). Different letters indicate significant differences at *P* < 0.05. Con indicates control treatment for each N nutrition, i.e., plants receiving non-water stress. FW: fresh weight; TBARS: thiobarbituric acid reactive substances

Endogenous plant NO production is mostly driven by NR and NOS activity [4]. Water stress increased NR activity in NO_3_^−^-treated roots, and this activity was higher at 24 h than it was at 3 h of water stress (Additional file 2: Figure S2a). The activity of NOS was also significantly elevated at 3 h of water stress, and significantly higher in the NH_4_^+^-treated than in the NO_3_^−^-treated roots (Additional file 2: Figure S2b). In contrast, water stress suppressed NOS activity in NO_3_^−^-treated roots at 24 h. Tungstate and L-NAME, which inhibit NR and NOS activities, respectively, were used to identify the origin of the early NO burst in the NH_4_^+^-treated roots [25]. Although L-NAME significantly inhibited endogenous NO production in NH_4_^+^-treated roots at 3 h of water stress, it had no significant effect in the NO_3_^−^-treated roots. At 24 h, the tungstate and L-NAME applications suppressed NO production in NO_3_^−^-treated roots, and tungstate had the stronger inhibitory effect. However, tungstate had no significant effect on NO production in the NH_4_^+^-treated roots (Fig. 5a, b).

The effect of SNP on the alleviation of water stress-induced root oxidative damage was reversed after pretreatment with 100 μM c-PTIO at 3 h. Application of the NOS inhibitor L-NAME significantly aggravated water stress-induced oxidative damage in NH_4_^+^-treated roots, and SNP application reversed the effect of the NOS inhibitor but not that of the NR inhibitor (Fig. 5c, d). For the NO_3_^−^-treated roots, the application of NR inhibitor or NOS inhibitor had no significant effect on root oxidative damage relative to the water stress treatment.

### Activities of antioxidative enzymes and nitrate/nitrite and arginine/citrulline metabolism

**Fig. 6:**
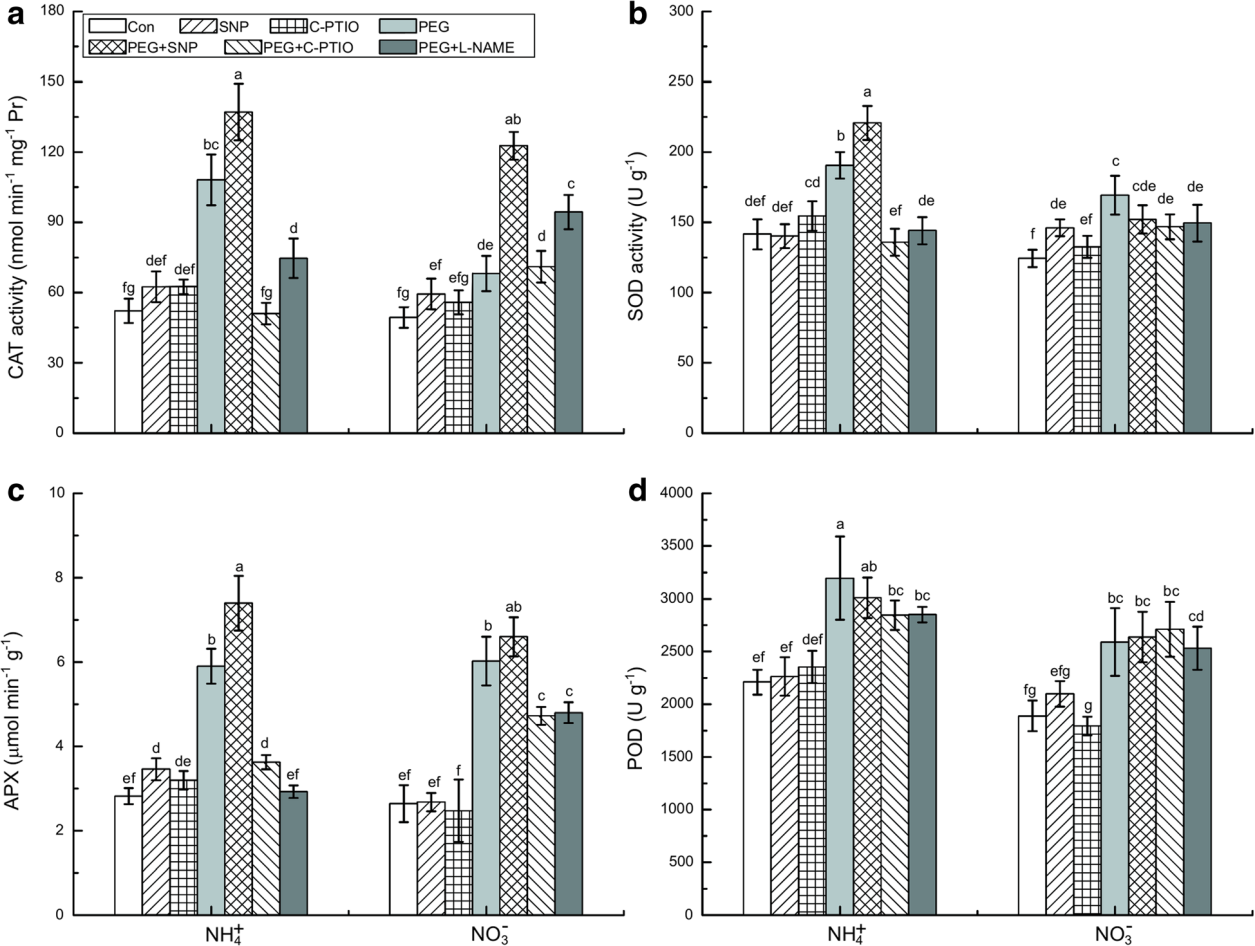
Effects of different treatments on antioxidant enzyme changes in rice seedlings under water stress. Roots were collected to assay CAT (**a**), SOD (**b**), APX (**c**), and POD (**d**) after 3 h of treatment with non-water stress (Con) or water stress. For the c-PTIO, PEG + c-PTIO, and PEG + L-NAME treatments, the rice seedlings were pretreated with NO scavenger (c-PTIO) or NOS inhibitor (100 μM L-NAME) for 3 h followed by non-water stress or water stress. Values represent means ± standard error (SE) (*n* = 6). Different letters indicate significant differences at *P* < 0.05. Con indicates control treatment for each N nutrition, i.e., plants receiving non-water stress

Water stress significantly enhanced the activities of the root antioxidant enzymes catalase (CAT), superoxide dismutase (SOD), ascorbate peroxidase (APX), and peroxidase (POD) by ~ 107 and 38%, 52 and 36%, 152 and 128%, and 45 and 37% in the NH_4_^+^-treated roots and NO_3_^−^-treated roots, respectively, compared to their Con roots (Fig. 6). While SNP application further increased CAT, SOD, and APX activities (Fig. 6a–c), these antioxidant enzymes were inhibited by the application of the NO scavenger c-PTIO and by the NOS inhibitor L-NAME in the NH_4_^+^-treated roots under water stress.

As NR and NOS activities increased in the NO_3_^−^-treated roots, water stress lowered the nitrate level in the NR pathway and the arginine level in the NOS pathway (Additional file 3: Figure S3a, b). Similarly, application of NR inhibitor and NOS inhibitor enhanced root nitrate and arginine contents, respectively. In the NH_4_^+^-treated roots, water stress significantly decreased the arginine level, indicating that arginine metabolism is relatively high. In this treatment, NR inhibitor had no significant effect on root arginine content. On the other hand, the NOS inhibitor suppressed arginine metabolism, and thus the NH_4_^+^-treated roots had higher arginine levels than Con roots (Additional file 3: Figure S3c). These results also indirectly indicate that the NO early production burst in NH_4_^+^-treated roots might originate from the NOS pathway.

## Discussion

Ample experimental evidence has demonstrated that NO is involved in plant abiotic stress [17]. However, to our knowledge, no detailed study has been conducted to evaluate the role of NO in drought acclimation in plants supplied with NO_3_^−^ or NH_4_^+^. In the present study, plant biomass, root N uptake rate, and leaf photosynthesis were reduced after 21 days of water stress relative to the non-water stress condition (Fig. 1). However, these reductions were less severe for seedlings receiving NH_4_^+^, suggesting that NH_4_^+^ nutrition can enhance drought tolerance in rice seedlings more effectively than NO_3_^−^ nutrition [2, 33]. Our study also demonstrated that, in the short term (48 h), endogenous NO production in response to water stress is time-dependent, varying according to water stress duration and N nutrition. This finding is consistent with those reported for other stressors [10, 25]. Early NO bursts were induced at 3 h of water stress in the roots of NH_4_^+^-treated seedlings but not in NO_3_^−^-treated seedlings. Thus, there might be significant differences between NH_4_^+^- and NO_3_^−^-supplied plants in terms of NO signal-mediated drought tolerance. In addition, accumulation of ROS, such as O_2_^.-^, OH^−^, and H_2_O_2_, and root oxidative damage were significantly lower in the NH_4_^+^-treated than in the NO_3_^−^-treated roots at 3 h of water stress (Fig. 4). Excessive accumulation of ROS damages cells and plasma membranes under the different abiotic stresses [11]. Whether the early NO burst in response to water stress observed in NH_4_^+^-supplied seedlings plays a crucial role in the plant antioxidant defense system needs further investigation, however.

The role of the early NO burst in the water stress tolerance of NH_4_^+^−/NO_3_^−^-supplied seedlings was confirmed using NO donors and scavengers. Our study demonstrated that NO donor induced NO in the NO_3_^−^-treated roots at 3 h but not at 24 h of water stress (Fig. 3a). Plant ROS accumulation and MDA and carbonyl levels under water stress were significantly alleviated after the application of the NO donor in both N nutritions (Fig. 3c, d). Nevertheless, the levels of these substances were higher in the NO_3_^−^-treated roots than in the NH_4_^+^-treated roots. Therefore, the NO production enhanced at 3 h by the exogenous NO donor can alleviate water stress-induced oxidative damage in the NO_3_^−^-treated roots. On the other hand, elimination of the early NO burst by NO scavengers like c-PTIO significantly aggravated water stress-induced oxidative damage (Fig. 3c, d). These results provide direct evidence that the early NO burst plays a crucial role in drought tolerance in NH_4_^+^-treated roots. Because the NH_4_^+^-supplied roots maintained a higher N uptake rate than NO_3_^−^-supplied roots under water stress (Fig. 1f), we hypothesized that the higher NH_4_^+^ uptake rate is beneficial for the early NO burst due to the NO production involved in root N metabolism [13, 34]. This NO burst can also be an active adaptation mechanism of plants to abiotic stress as, in addition to drought stress, it has been reported to occur repeatedly in plants challenged by pathogens [35], metal toxicity [9, 25], and cold stress [36].

Our study further demonstrated that an early NO burst improves plant drought tolerance by enhancing the antioxidant defense system of the root. Elevated plant antioxidant enzyme activities and gene expression levels in response to water stress have been widely demonstrated [12, 14, 18]. In the present study, the tips of the NO_3_^−^-treated roots presented more serious water stress-induced oxidative damage (due to the excessive production of O_2_^.-^, OH^−^, and H_2_O_2_) than those of the NH_4_^+^-treated roots (Figs. 2–4). In contrast, NH_4_^+^-supplied roots maintained relatively higher antioxidant enzyme (CAT, SOD, and APX) activity levels to catalyze O_2_^.-^ and H_2_O_2_ decomposition (Fig. 6). It has been demonstrated that there is significant crosstalk between NO and ROS in plants. The antioxidant function of NO was explained by its ability to reduce H_2_O_2_ and lipid peroxidation, and induce antioxidant gene expression and enzyme activity [1, 14]. Our results showed that enhanced NO level and antioxidant enzyme activities (CAT and SOD) were significantly and simultaneously increased after NO donor application in NO_3_^−^-treated roots, thereby reducing ROS concentration and oxidative damage (Figs. 3, 6). The early NO burst observed in NH_4_^+^-treated roots can enhance antioxidant enzyme activity and ROS accumulation (O_2_^.-^, OH^−^, and H_2_O_2_). These results were confirmed by subsequent experimentation in which the application of NO scavenger significantly suppressed SOD and CAT in NH_4_^+^-treated roots. Thus, drought tolerance in the NH_4_^+^-treated roots might be associated with the NO-induced up-regulation of antioxidant enzymes and down-regulation of ROS accumulation.

Nitric oxide can also serve as a source of reactive nitrogen species (RNS). Overaccumulation of RNS under abiotic stress can cause tyrosine nitration and inactivate proteins like CAT, manganese-dependent (Mn-)SOD, and GR as well as the peroxidative activity of cytochrome *c* [37, 38]. Our results showed that NO_3_^−^-supplied plants had more severe oxidative damage and accumulated extremely high NO levels after 24 h of water stress (Fig. 3). This latent NO production can be partially alleviated by replenishing the early NO burst at 3 h with SNP. These results indicate that both ROS and RNS metabolism participate in the water stress response. High NO accumulation in the NO_3_^−^-treated roots likely caused the nitrosative stress at 24 h, which also damaged root redox balance. A similar phenomenon was described in plants subjected to cold [39], salinity [40], and drought [7] stresses. Because NO competes with oxygen for cytochrome *c* oxidase binding (Complex IV), it affects both the respiratory chain and oxidative phosphorylation [41, 42]. Thus, under water stress, the higher NO production in the NO_3_^−^-treated roots than in the NH_4_^+^-treated roots could aggravate respiratory inhibition and induce greater oxidative damage.

**Fig. 7:**
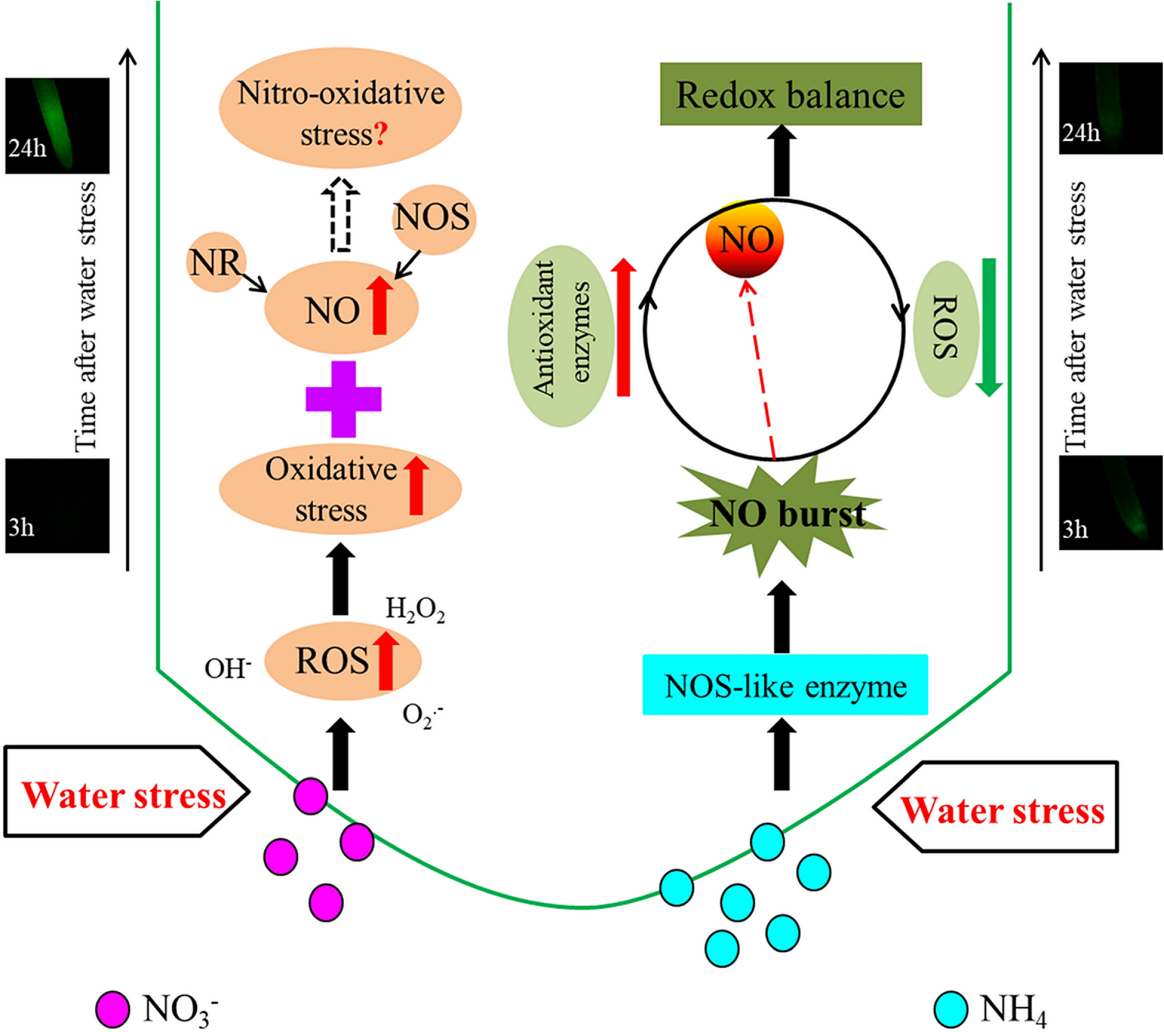
Schematic illustration of a proposed model for the different responses of early NO production and its effects on the defense response of rice to water stress. In the roots of NH_4_^+^-supplied rice, the NOS-mediated early NO burst (3 h) significantly enhanced plant antioxidant defense by reducing ROS accumulation and enhancing antioxidant enzyme activity; the relative lower NO production after 24 h of water stress in comparison to NO_3_^−^-supplied rice also helped maintaining the redox balance in root cells, thus enhancing their drought tolerance. In the roots of NO_3_^−^-supplied rice, ROS accumulation and oxidative damage induced by 3 h of water stress were significantly higher than that in NH_4_^+^-supplied rice. High NO accumulation in the NO_3_^−^-treated roots likely caused the nitrosative stress at 24 h of water stress. A combined effect of oxidative and nitrification stresses might have led to the weak resistance to water stress in NO_3_^−^-supplied rice. NR, nitrate reductase. Red arrows represent increase; green arrows represent decrease. Black solid arrows represent defined pathways, dotted arrows represent undefined pathway

Our investigation further suggests that the early NO burst in NH_4_^+^-treated roots is mainly mediated by NOS at the early stages of water stress. Nitrate reductase-mediated NO generation is known to occur under water deficit [19, 43]. Drought-induced NO generation by NOS-like enzymes in plants has also been demonstrated but this NO production pathway varies significantly with species, tissue type, and plant growth conditions [29, 30]. For the NH_4_^+^-treated roots, both NOS activity and NO production increased simultaneously at 3 h of water stress, whereas the application of the NOS inhibitor completely repressed NO synthesis at this time point. The NOS inhibitor also aggravated water stress-induced membrane lipid peroxidation and oxidative protein damage, indicating that some NOS-associated proteins may play an important role in NO-mediated drought-protective responses [8, 23]. In contrast, the NR inhibitor did not significantly affect NO production or membrane lipid peroxidation. The aggravation of lipid peroxidation by L-NAME may have been the result of the alteration of the NOS-mediated early NO burst. In NO_3_^−^-treated roots, water stress enhanced NR activity significantly more than NOS activity at 24 h. However, separate NR inhibitor and NOS inhibitor application only partially suppressed NO production. The NO produced by the NR pathway might therefore play an important role in later NO production (24 h), consistent with previous reports [18, 19]. Although several studies support the arginine-dependent NO production model in higher plants, the identification of genes encoding NOS in such plants is still up for debate [28]. For this reason, the nitrate/nitrite and arginine/citrulline levels in the NR and NOS pathways, respectively, were determined. It was found that water stress significantly increased NOS activity and accelerated the conversion of arginine to citrulline in both N nutritions. However, in relation to the Con roots the arginine content was significantly enhanced in the NH_4_^+^-treated roots after application of the NOS inhibitor. These results provide additional evidence that the early NO burst in NH_4_^+^-treated roots is mainly mediated by NOS (Fig. 7).

## Conclusions

Our study demonstrated that the early NO burst in NH_4_^+^-treated rice roots significantly enhanced plant antioxidant defense by reducing ROS accumulation and enhancing the activities of antioxidant enzymes, thereby increasing plants’ acclimation to water stress. The early NO burst that occurs in response to water stress may be triggered by NOS-like enzymes in root. Our results provide new insight into how NO-signaling molecules modulate drought tolerance in NH_4_^+^-supplied rice plants. However, in future the definite evidencefor this pathway provided by genetic and molecular techniques still need to be developed to achieve the target-specific editing of NO biosynthetic and signaling pathways under water deficits.

## Methods

### Plant material and growth conditions

Rice (*Oryza sativa* L. ‘Zhongzheyou No. 1’ hybrid *indica*) seedlings, obtained from the China National Rice Research Institute, were grown hydroponically in a greenhouse. Seeds were sterilized in 1% (*v*/v) sodium hypochlorite solution. After germination, seeds were transferred to a 0.5 mM CaCl_2_ solution (pH 5.5). Three days later, the seedlings were transferred to 1.5-L black plastic pots containing a solution with the following composition: NH_4_NO_3_ (0.5 mM), NaH_2_PO_4_·2H_2_O (0.18 mM), KCl (0.18 mM), CaCl_2_ (0.36 mM), MgSO_4_·7H_2_O (0.6 mM), MnCl_2_·4H_2_O (9 μM), Na_2_MoO_4_·4H_2_O (0.1 μM), H_3_BO_3_ (10 μM), ZnSO_4_·7H_2_O (0.7 μM), CuSO_4_ (0.3 μM), and FeSO_4_·7H_2_O–EDTA (ethylenediaminetetraacetic acid) (20 μM). All experiments were performed in a controlled growth room under the following conditions: 14/10 h light/dark photoperiod, 400 μmol·m^− 2^·s^− 1^ light intensity, 28/23 °C during the day and night, respectively, and 60% relative humidity. The solution pH was adjusted to 5.5 with 5 mM 2-(*N*-morpholino)ethanesulfonic acid (MES). The solution was replaced every 3 days.

After 6 days, seedlings of similar size were cultivated under the four following treatments: 1 mM NO_3_^−^, 1 mM NO_3_^−^ + 10% PEG (PEG-6000), 1 mM NH_4_^+^ and 1 mM NH_4_^+^ + 10% PEG-6000. Water stress was induced by adding 10% PEG-6000. Eight treatments were performed in the NO donor (SNP) experiments: NH_4_^+^, NH_4_^+^ + SNP, NH_4_^+^ + PEG-6000, NH_4_^+^ + PEG-6000 + SNP, NO_3_^−^, NO_3_^−^ + SNP, NO_3_^−^ + PEG-6000, and NO_3_^−^ + PEG-6000 + SNP. The final SNP concentration was 20 μM. Each treatment had six replicates. For each N nutrition, plants cultivated under non-water stress condition were defined as the control (Con) relative to the water stress (PEG) condition.

To determine the role of NO in the plant antioxidant defense system under water stress, rice seedlings supplied with 1 mM NO_3_^−^ or 1 mM NH_4_^+^ solution were pretreated with 100 μΜ c-PTIO (as NO scavenger) for 3 h, and then subjected to non-water stress (Con treatment) or water stress (PEG) for 24 h under the same condition as those described above. Each treatment had six replicates.

To investigate the origin of the endogenous NO produced under water stress, rice seedlings supplied with 1 mM NO_3_^−^ or 1 mM NH_4_^+^ solution were pretreated with the NR inhibitor (tungstate, 100 μΜ) or NOS inhibitor (L-NAME, 100 μΜ) for 3 h, and then subjected to non-water stress (Con) or water stress for 24 h under the same conditions as described above. There were eight treatments for each N nutrition: tungstate, L-NAME, tungstate + SNP, PEG-6000 + tungstate, PEG-6000 + tungstate + SNP, L-NAME + SNP, PEG-6000 + L-NAME, and PEG-6000 + L-NAME + SNP. Each treatment had six replicates.

### Determination of NO and ONOO^−^contents

The DAF-FM DA probe was used to determine the endogenous root NO level [25]. Root tips (1 cm) were incubated with 10 μM DAF-FM DA in the dark for 30 min, washed three times with 20 mM HEPES–KOH (pH 7.4) to remove excess fluorescence, and then observed and photographed under a Nikon Eclipse 80i fluorescence microscope (Nikon, Tokyo, Japan; excitation filter 460–500 nm, dichroic mirror 505 nm, barrier filter 510–560). The relative fluorescence intensity was measured with Photoshop v. 7.0 (Adobe Systems, Mountain View, CA, USA).

Root endogenous ONOO^−^ was determined using the aminophenylfluorescein (APF) probe method [44]. Root tips were incubated with 10 μM APF dissolved in 10 mM Tris–HCl (pH 7.4) in the dark for 60 min, and then washed three times with 10 mM Tris–HCl. Fluorescence images and relative fluorescence intensities were analyzed as described above for NO.

### Histochemical analyses

Lipid peroxidation and root cell death were detected histochemically with Schiff’s reagent and Evans blue [45]. Root tips were incubated in Schiff’s reagent for 20 min and washed by three consecutive immersions in 0.5% (*w*/*v*) K_2_O_3_S solution. A red/purple endpoint indicated the presence of aldehydes generated by lipid peroxidation. Roots were also washed by performing three serial immersions in distilled water, then incubated in 0.25% (w/v) Evans blue for 15 min, and finally washed three times with distilled water. Roots stained with Schiff’s reagent and Evans blue were immediately photographed under a Leica S6E stereomicroscope (Leica, Solms, Germany).

The oxidative damage level, specifically expressed as membrane lipid peroxidation and protein oxidative damage, was estimated by measuring the concentrations of MDA and carbonyl group with 2,4-dinitrophenylhydrazine (DNPH) [46].

### Determination of ROS contents

Root O_2_^.-^ content was estimated using the method described in Liu et al. [47] with some modifications: about 0.15 g fresh root was powdered with 2 mL of 65 mM phosphate buffer saline (PBS, pH 7.8) in a pre-cooled mortar, and centrifuged at 5000 *g* and 4 °C for 10 min. Then, 0.9 mL of 65 mM PBS (pH 7.8) and 0.1 mL of 10 mM hydroxylammonium chloride were added to 1 mL of the root extract supernatant, thoroughly mixed, and left to react for 25 min. After this period, 1 mL of 1% (*w*/*v*) sulfanilamide and 1 mL of 0.02% (w/v) *N*-(1-naphthyl)-ethylenediaminedihydrochloride were added to 1 mL of root extract solution and left to react for 30 min. Absorbance was then measured at 540 nm.

Root H_2_O_2_ content was determined by the photocolorimetric method [48]: ~ 0.15 g fresh root was powdered with 2 mL acetone in a pre-cooled mortar, and centrifuged at 5000 *g* and 4 °C for 10 min. Then, 0.1 mL of 5% (w/v) TiSO_4_ and 0.1 mL pre-cooled ammonium hydroxide were added to 1 mL of the root extract supernatant, which was re-centrifuged at 5000 *g* for 10 min. The supernatant was discarded and the sediment was re-dissolved in 4 mL of 2 M H_2_SO_4_. The absorbance of the root extract solution was measured at 415 nm.

Root OH^−^ content was analyzed by the methods described in a previous study [49]: ~ 0.1 g fresh root was powdered with 3 mL of 50 mM PBS (pH 7.0) in a mortar, and centrifuged at 10,000 *g* and 4 °C for 10 min. Then, 1.0 mL of 25 mM PBS (pH 7.0) containing 5 mM 2-deoxy-*D*-ribose and 0.2 mM NADH were added to 1 mL of the root extract supernatant, completely blended, and left to react for 60 min at 35 °C in the dark. Following this incubation, 1 mL of 1% (w/v) thiobarbituric acid and 1 mL glacial acetic acid were added to the filtrate. The mixture was heated to 100 °C for 30 min and then placed on ice for 20 min. The absorbance of the root extract solution was then measured at 532 nm, and the OH^−^ content was inferred from the production of MDA.

### Determination of enzyme activities

Fresh rice root samples (0.5 g) were homogenized in 5 mL of 10 mM phosphate buffer (pH 7.0) containing 4% (w/v) polyvinylpyrrolidone and 1 mM ethylenediaminetetraacetic acid. The supernatant was used as crude enzyme solution and collected by centrifugation at 12,000 *g* and 4 °C for 15 min. The activities of SOD, CAT, APX, and POD were estimated using the photocolorimetric methods described in Jiang and Zhang [11], and Sachadyn-Krol et al. [50].

Root NR and NOS activities were assayed using the methods described in previous studies [25, 26], with some modifications. Briefly, total protein was extracted using a buffer containing 100 mM HEPES–KOH (pH 7.5), 1 mM EDTA, 10% (*v*/v) glycerol, 5 mM 1,4-dithiothreitol (DTT), 0.5 mM phenylmethylsulfonyl fluoride, 0.1% Triton X-100 (v/v), 1% polyvinylpyrrolidone (PVP), and 20 μM flavin adenine dinucleotide. The supernatant was collected by centrifugation at 12,000 *g* and 4 °C for 20 min, and then used to determine the NR and NOS activities at 520 nm and 340 nm, respectively.

Specifically, the activity of NR was measured immediately by mixing 250 μL of supernatant with 250 μL pre-warmed (25 °C) assay buffer containing 50 mM HEPES–KOH (pH 7.5), 10 mM MgCl_2_, 1 mM DTT, 2 mM KNO_3_ and 200 μM NADH. The reaction was started by adding assay buffer, incubated at 30 °C for 30 min and then stopped by adding 50 μL 0.5 M Zn-acetate. The nitrite produced was measured colorimetrically at 540 nm after adding 1 mlof 1% sulfanilamide in 3 M HCl plus 1 mL of 0.02% *N*-(1-naphthyl)ethylenediamine in 0.2 M HCl. NOS activity was detected in 1 mL of reaction mixture containing 100 mM phosphate buffer (pH 7.0), 1 mM laevo-arginine(L-Arg), 2 mM MgCl_2_, 0.3 mM CaCl_2_, 4 μM BH_4_, 1 μM FAD, 1 μM flavin mononucleotide (FMN), 0.2 mM DTT, 0.2 mM NADPH, and 200 μL of protein extract. The decrease in absorbance as a result of NADPH consumption was determined at 340 nm for 5 min. NOS activity was calculated using the extinction coefficient of NADPH (ɛ = 6.22 mM^− 1^·cm^− 1^).

### Determination of arginine and citrulline

Arginine and citrulline contents were estimated using the method described in Salazar et al. [51]. Briefly, 1.0 g root samples were frozen in liquid N_2_ and extracted with 4 mL 80% (v/v) methanol, and then centrifuged at 10,000 *g* and 4 °C for 5 min. The supernatant was then used in derivatization and reaction processes. Serial concentrations of amino acid standards were prepared as described above for the derivatizing reagent, and the derivatizing samples were used to determine the arginine and citrulline contents using liquid chromatography/electrospray ionization tandem mass spectroscopy (LC-ESI-MS).

### Statistical analyses

All experiments conducted in this study were performed in six replicates, at least. All data, expressed as means ± standard error (SE), were processed in SPSS v. 13.0 (IBM Corp., Armonk, NY, USA). The Least Significant Difference (LSD) test was used to determine statistical significant differences among the treatments (*P* < 0.05). Figures were drawn in Origin v. 8.0 (OriginLab Corporation, Northampton, MA, USA).

## Additional files

Additional file 1: **Figure S1.** Effect of exogenous NO donor (SNP) on root oxidative damage under water stress. Rice roots were exposed to mixed N (NH_4_^+^ + NO_3_^−^) nutrient solution containing 0 μM, 5 μM, 10 μM, 20 μM, 40 μM, 80 μM, or 100 μM SNP either with or without 10% PEG for 48 h. The contents of MDA representing lipid peroxidation (a) and carbonyl group (b) in rice seedling roots were determined. Values represent means ± SE (*n* = 6). Different letters indicate significant differences at *P* < 0.05. Con indicates control treatment for each N nutrition, i.e., plants receiving non-water stress. FW: fresh weight; TBARS: thiobarbituric acid reactive substances. (TIF 71 kb)

Additional file 2: **Figure S2.** Effect of water stress on NR (a) and NOS (b) in roots. Roots were collected for the NR and NOS assays after 3 h and 24 h of water stress, respectively. Values represent means ± SE (*n* = 6). Different letters indicate significant differences at *P* < 0.05. Con indicates control treatment for each N nutrition, i.e., plants receiving non-water stress. (TIF 41 kb)

Additional file 3: **Figure S3.** Effect of exogenous NR inhibitor (tungstate) and NOS inhibitor (L-NAME) on the related compounds in NR-mediated and NOS-mediated NO pathways. (a) Levels of nitrate and nitrite in NO_3_^−^-treated roots. (b) Levels of arginine and citrulline in NO_3_^−^-treated roots. (c) Levels of arginine and citrulline in NH_4_^+^-treated roots. For the PEG + tungstate and PEG + L-NAME treatments, the rice seedlings were pretreated with NR inhibitor (100 μM tungstate) or NOS inhibitor (100 μM L-NAME) for 3 h, followed by non-water stress (Con) or water stress treatment. Values represent means ± SE (*n* = 6). Different letters indicate significant differences at *P* < 0.05. (TIF 69 kb)

Additional file 4: **Method S1.** Determination of leaf photosynthesis, root N uptake rate, and root nitrate and nitrite contents in rice seedlings after 21 days of non-water stress (Con) or water stress (PEG) treatment. (DOCX 14 kb)

## Abbreviations

ABA: Abscisic acid
APF: Aminophenylfluorescein
APX: Ascorbate peroxidase
CAT: Catalase
c-PTIO: 2-(4-carboxyphenyl)-4,4,5,5-tetramethylimidazoline-1-oxyl-3-oxide
DAF-FM DA: Diaminofluorescein-FM diacetate
DNPH: 2,4-dinitrophenylhydrazine
DTT: 1,4-dithiothreitol
EDTA: Ethylenediaminetetraacetic acid
FAD: Flavin adenine dinucleotide
FMN: Flavin mononucleotide
GR: Glutathione reductase
GSNO: *S*-nitrosoglutathione
L-Arg: Leavo-arghinine
LC-ESI-MS: Liquid chromatography/electrospray ionization tandem mass spectroscopy
L-NAME: N(G)-nitro-*L*-arginine methyl ester
MDA: Malondialdehyde
MES: 2-(*N*-morpholino)ethanesulfonic acid
NADPH: Nicotinamide adenine dinucleotide phosphate
NO: Nitric oxide
NOS: Nitric oxide synthase
NR: Nitrate reductase
ONOO^−^: Peroxynitrite
PBS: Phosphate buffer saline
PEG: Polyethylene glycol
*P*_n_: Net photosynthetic rate
POD: Peroxidase
PVP: Polyvinylpyrrolidone
RNS: Reactive nitrogen species
ROS: Reactive oxygen species
SNP: Sodium nitroprusside
SOD: Superoxide dismutase
