# Supplementary figures and images for: Nitric oxide synthase-mediated early nitric oxide burst alleviates water stress-induced oxidative damage in ammonium-supplied rice roots
Source: BMC Plant Biol. 2019 Mar 20;19:108. doi: 10.1186/s12870-019-1721-2 (PMC6425712; doi:10.1186/s12870-019-1721-2)

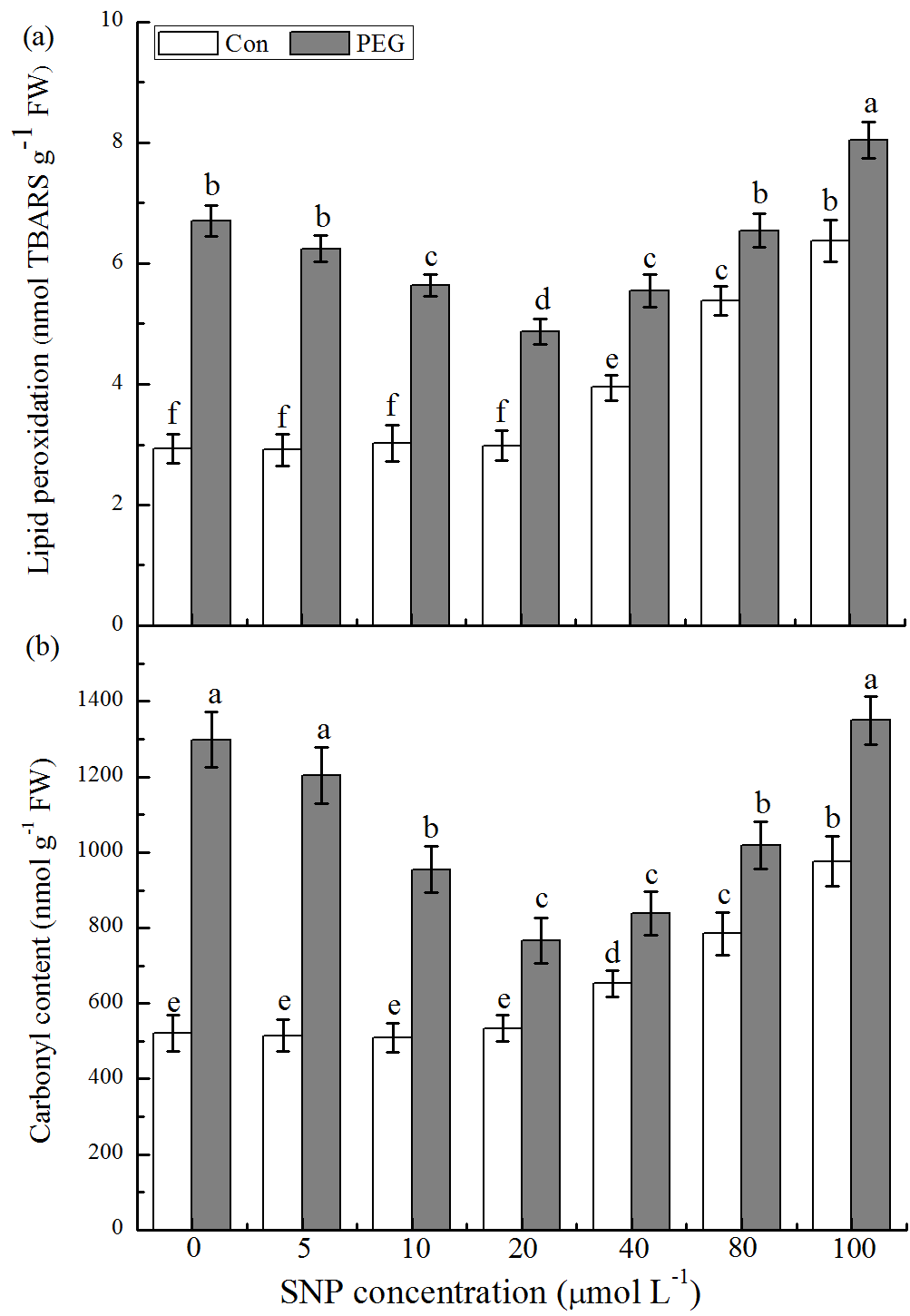

Supplement: Supplementary file 1 — Figure S1. Effect of exogenous NO donor (SNP) on root oxidative damage under water stress. Rice roots were exposed to mixed N (NH4+ + NO3−) nutrient solution containing 0 μM, 5 μM, 10 μM, 20 μM, 40 μM, 80 μM, or 100 μM SNP either with or without 10% PEG for 48 h. The contents of MDA representing lipid peroxidation (a) and carbonyl group (b) in rice seedling roots were determined. Values represent means ± SE (n = 6). Different letters indicate significant differences at P < 0.05. Con indicates control treatment for each N nutrition, i.e., plants receiving non-water stress. FW: fresh weight; TBARS: thiobarbituric acid reactive substances. (TIF 71 kb) [file 12870_2019_1721_MOESM1_ESM.tif]

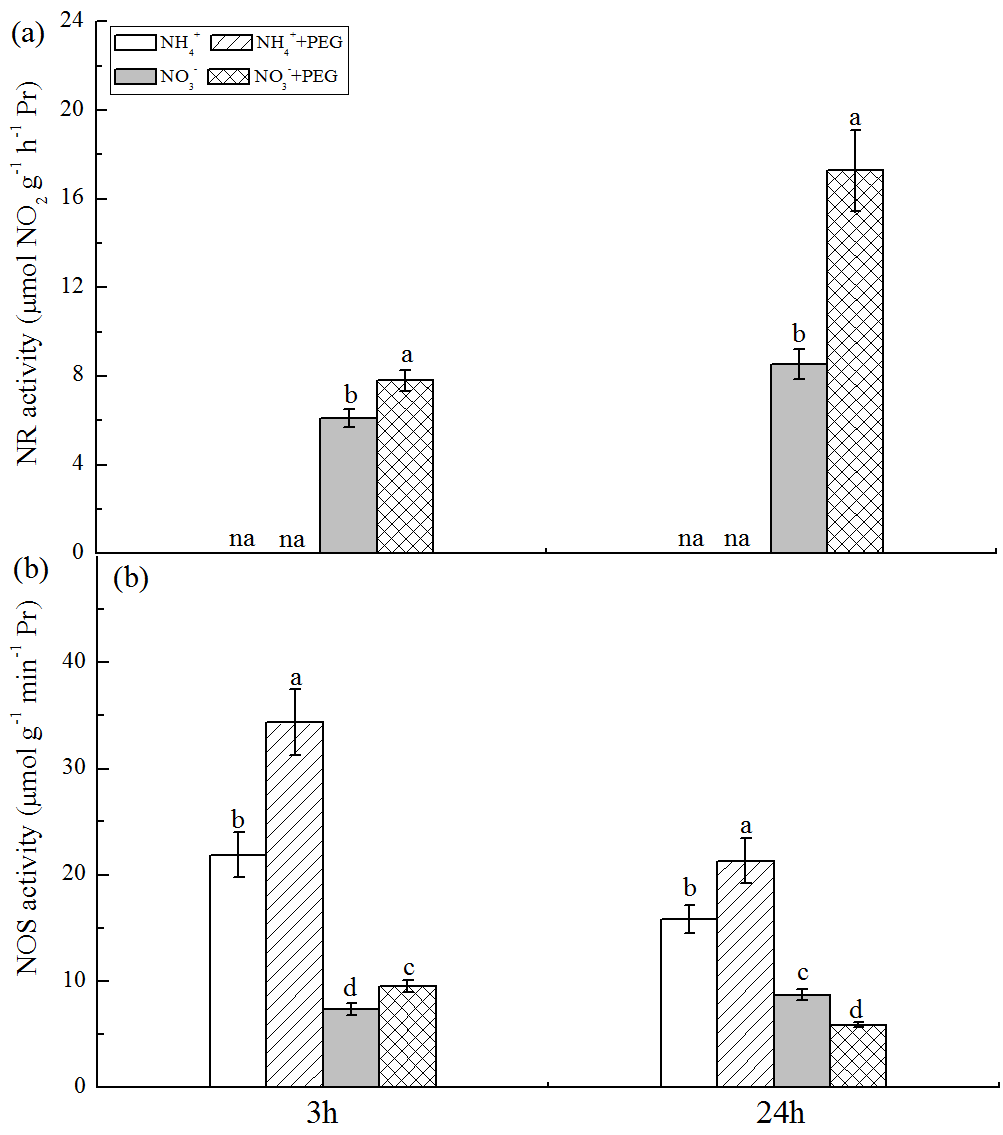

Supplement: Supplementary file 2 — Figure S2. Effect of water stress on NR (a) and NOS (b) in roots. Roots were collected for the NR and NOS assays after 3 h and 24 h of water stress, respectively. Values represent means ± SE (n = 6). Different letters indicate significant differences at P < 0.05. Con indicates control treatment for each N nutrition, i.e., plants receiving non-water stress. (TIF 41 kb) [file 12870_2019_1721_MOESM2_ESM.tif]

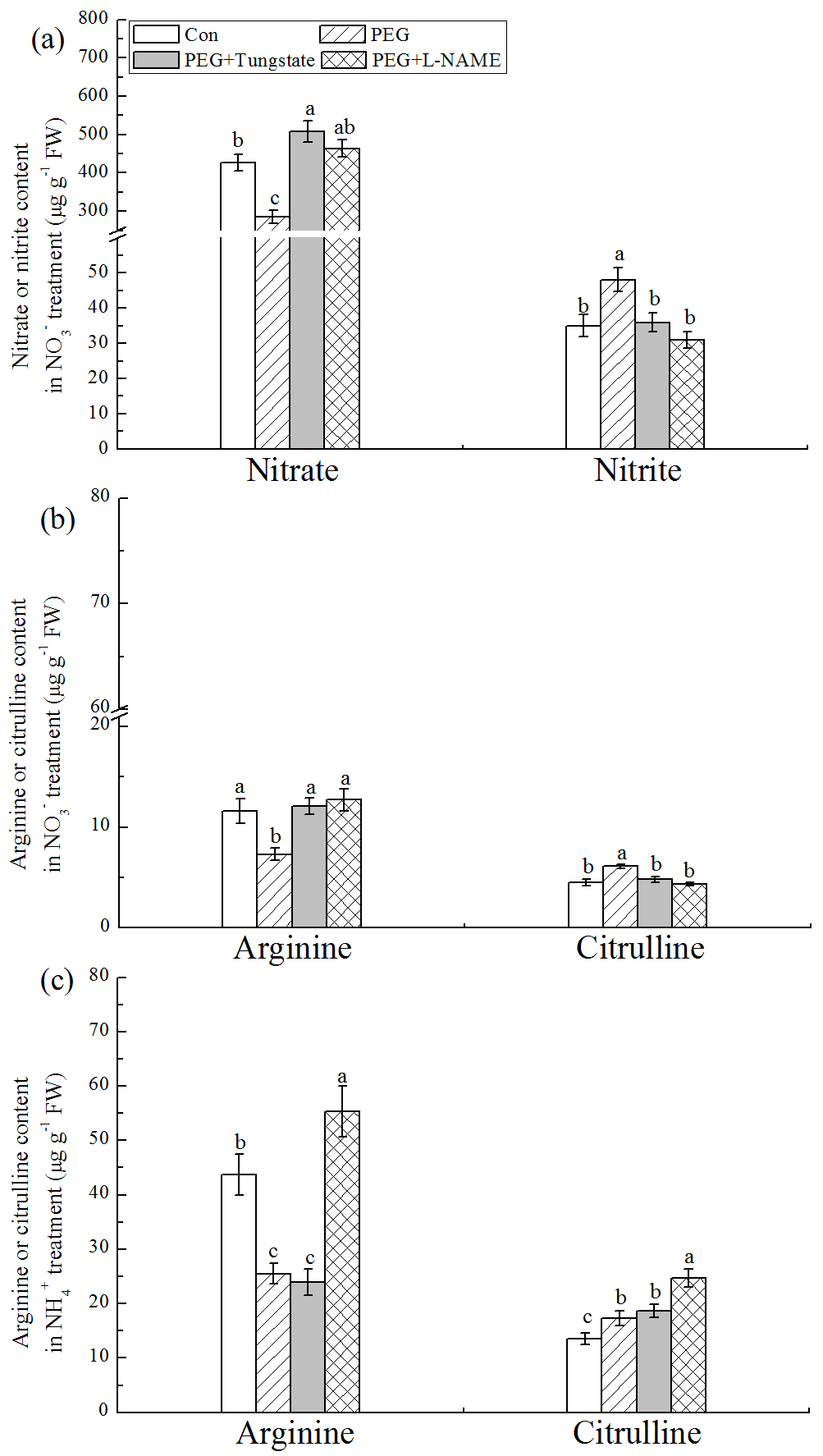

Supplement: Supplementary file 3 — Figure S3. Effect of exogenous NR inhibitor (tungstate) and NOS inhibitor (L-NAME) on the related compounds in NR-mediated and NOS-mediated NO pathways. (a) Levels of nitrate and nitrite in NO3−-treated roots. (b) Levels of arginine and citrulline in NO3−-treated roots. (c) Levels of arginine and citrulline in NH4+-treated roots. For the PEG + tungstate and PEG + L-NAME treatments, the rice seedlings were pretreated with NR inhibitor (100 μM tungstate) or NOS inhibitor (100 μM L-NAME) for 3 h, followed by non-water stress (Con) or water stress treatment. Values represent means ± SE (n = 6). Different letters indicate significant differences at P < 0.05. (TIF 69 kb) [file 12870_2019_1721_MOESM3_ESM.tif]
