# Supplementary material for: Nitric oxide synthase-mediated early nitric oxide burst alleviates water stress-induced oxidative damage in ammonium-supplied rice roots
Source: BMC Plant Biol. 2019 Mar 20;19:108. doi: 10.1186/s12870-019-1721-2 (PMC6425712; doi:10.1186/s12870-019-1721-2)
Supplement: Supplementary file 4 — Method S1. Determination of leaf photosynthesis, root N uptake rate, and root nitrate and nitrite contents in rice seedlings after 21 days of non-water stress (Con) or water stress (PEG) treatment. (DOCX 14 kb) [file 12870_2019_1721_MOESM4_ESM.docx]

**Method S1.** Determination of leaf photosynthesis, root N uptake rate, and root nitrate and nitrite contents in rice seedlings after 21 days of non-water stress (Con) or water stress (PEG) treatment.

*Determination of leaf photosynthesis*

After 21 days of non-water stress or water stress cultivation, net leaf photosynthesis was measured on sunny days at ambient CO_2_ concentration (*C*_a_; ~390 mmol mol^-1^) using a portable photosynthesis system (Li-6400XT; LI-COR Biosciences, Lincoln, NE, USA). Leaf temperature was maintained at 28°C. Photosynthetic photon flux density (PPFD) was 1,000 μmol·m^-2^·s^-1^ and relative humidity was 60–70%.

*Determination of root ^15^N uptake rate*

The same six replicates per treatment (NO_3_^-^, NO_3_^-^ + PEG, NH_4_^+^, and NH_4_^+^ + PEG) were used to measure root N uptake. First, the rice seedlings were treated with deionized water for 4 h to achieve the nutrient starvation condition. Then, root N uptake rates were determined for the four treatments from the absorption of 1 mM ^15^N-labeled substrates (50 atom% ^15^NO_3_^-^ and 50 atom% ^15^NH_4_^+^). The uptake solution also contained the nutrients listed in the subsection “Plant material and growth conditions”. After 6 h of incubation, the root systems were excised, washed with 50 mM CaCl_2_, and rinsed with deionized water. The rice shoots (leaves and stems) and the roots were stored at -80°C and freeze-dried (Labconco FreeZone Freeze Dry System, Kansas City, MO, USA). Their total biomass was determined by weighing. The ^15^N enrichment and N content in the dried roots were determined with Finnigan Tracer MAT-271 (Thermo Fisher Scientific, Waltham, MA, USA).

*Determination of root nitrate and nitrite*

Nitrate quantitation in plant tissue was performed as follows: ~1.0 g fresh root samples were extracted with 10 mL deionized water in a boiling water bath for 30 min. Then, 0.1 mL of the root extract supernatant was mixed thoroughly with 0.4 mL of 5% (w/v) salicylic acid in concentrated H_2_SO_4_ and incubated at 25°C for 20 min. A total of 9.5 mL of 2 M NaOH was added to increase the pH to > 12. The NO_3_^-^ concentration was measured at 410 nm.

The NO_2_^-^ concentration was determined as follows: ~100 mg fresh root samples were extracted in 1 mL extraction solution containing 50 mM Tris–HCl, 5 mM cysteine, and 2 mM EDTA. The homogenate was centrifuged at 10,000 *g* at 4°C for 20 min. NO_2_^-^ was measured at 540 nm after mixing 500 mL root extract supernatant with 250 mL of 1% (w/v) sulfanilamide and 250 mL of 0.02% (w/v) *N*-(1-naphthyl) ethylenediaminedihydrochloride.
